# Therapeutic Drug Monitoring and Adverse Drug Reactions: From Signal Detection to Prevention

**DOI:** 10.3390/ph19081308

**Published:** 2026-08-19

**Authors:** Engi Algharably, Ursula Gundert-Remy

**Affiliations:** Charité–Universitätsmedizin Berlin, Corporate Member of Freie Universität Berlin and Humboldt-Universität zu Berlin, Institute of Clinical Pharmacology and Toxicology, Charitéplatz 1, 10117 Berlin, Germany; ursula.gundert-remy@charite.de

Optimizing pharmacotherapy is a central goal of clinical pharmacology. Over recent decades, remarkable therapeutic advances have expanded the range of effective treatments available to patients; yet, this progress has also increased the complexity of medication safety. The clinical arena now encompasses diverse biologicals, targeted therapies, immunomodulators, and combination regimens. However, real-world patient populations receiving these drugs after marketing approval are far more heterogeneous than the selective cohorts studied in pre-approval trials. The efficacy and toxicity of these agents are substantially driven by patient-specific factors, including age, genetics, comorbidities, organ function, and concomitant medications. Within this context, adverse drug reactions (ADRs) present a persistent clinical challenge: while some ADRs are predictable and concentration-related, others are rare, and only become detectable after widespread clinical use; furthermore, ADRs with delayed onset often go undetected during the limited timeframe of pre-approval trials. The underlying mechanisms are similarly diverse, spanning immune-mediated and genetically based propensity, among others [1,2,3].

Investigating ADRs requires a broad methodological framework that integrates various disciplines to advance our understanding of drug toxicity, which encompasses knowledge from pharmacovigilance, pharmacoepidemiology, pharmacogenetics, mechanistic research, and pharmacokinetic (PK) modeling. Within this paradigm, therapeutic drug monitoring (TDM) makes a particularly vital contribution. Traditionally used to guide dosing of narrow-therapeutic-index drugs with high interindividual variability, TDM offers an important tool for reducing preventable toxicity by measuring concentrations or area-under-the concentration-time curves (AUCs). TDM bridges population-level safety data with individualized toxicity prevention.

The Special Issue “Therapeutic Drug Monitoring and Adverse Drug Reactions” brings together this range of approaches. Its contributions illustrate how ADR research can move from the detection of safety signals, to the identification of susceptible patients and mechanisms, and finally to individualized strategies that may prevent or reduce harm. Within the scope of ADR detection, several contributions focused on post-marketing pharmacovigilance and the detection of ADR signals in large real-world databases. These studies utilized spontaneous reporting systems such as the FDA Adverse Event Reporting System (FAERS), EudraVigilance, VigiBase, and national pharmacovigilance databases to identify potential safety concerns across a wide range of therapeutic areas. For canakinumab, a humanized anti-IL-1ß monoclonal antibody, infections were the most relevant serious adverse event and overweight patients were the population under greatest risk (Contribution Thaibah1). Thaibah et al. (Contribution 2) identified young male users of finasteride, a 5-alpha-reductase inhibitor, as the population at risk for suicidal attempts by using disproportionality methods. Co-prescribing SSRIs and NSAIDs in patients with depression was associated with higher risk of gastrointestinal bleeding, thrombocytopenia, and acute kidney injury, with some combinations increasing the risk more than others (Contribution 3); for example, citalopram with propionic acid derivatives and paroxetine with NSAIDs show significant drug–drug interaction signals. The findings do not prove causation, but they highlight specific combinations that repeatedly show disproportionate reporting signals.

All three publications based their analysis on the data extracted from the FEARS database. Similar approaches were applied to investigate neuropsychiatric events associated with ibuprofen and bullous pemphigoid with DPP-4 inhibitors in the EudraVigilance database (Contributions 4 and 5). These large databases offer the statistical power necessary to capture both high-frequency adverse reactions and rare safety signals that smaller datasets or pre-approval trials lack the power to identify. However, spontaneous reporting databases have inherent limitations including underreporting, reporting bias, missing clinical detail, duplicate reports and lack of denominator data. In addition, databases are not curated to facilitate an overarching analysis across multiple databases. Notably, these publications were based on a specific database and none evaluated whether signals identified in one dataset can be replicated in another. Disproportionality signals should not be interpreted as proof of causality; instead, they represent initial alerts that require further studies to confirm the “signals”, such as cohort studies, mechanistic research, or prospective clinical monitoring. As such, recommendations made by some of the authors might be premature because a signal is not clear proof. However, if the results are carefully interpreted, taking into account the uncertainty of the reported cases in the database, the analysis is still of value at least as a stimulus to further investigate the issue with a differing methodological approach.

A second group of contributions moves from signal detection toward explanation. ADRs do not occur uniformly because interindividual variability in PK and pharmacodynamics (PD) renders certain individuals more susceptible to toxicity. Pharmacogenetic aspects play an important role influencing the PK—mainly the metabolism—and the PD of a variety of drugs. A recent publication elaborated on this aspect, in particular for Arab countries [4]. Pharmacogenetic studies in this Special Issue illustrate this point. Whereas most of the investigations address the association between GST polymorphism and drug-induced ADR [5], the authors of Contribution 6 studied the association of GST polymorphism with metabolic syndrome in patients treated with antipsychotic drugs for which obesity and obesity-related disease are known ADRs. The study highlighted that the *GSTP1* variant significantly influences this metabolic risk, confirming that oxidative stress and detoxification pathways contribute to antipsychotic-induced toxicity. Perrez-Gomez et al. (Contribution 7) used TDM and pharmacogenetic biomarkers of ibrutinib-metabolizing enzymes and transporters to explain variability in response and toxicity in chronic lymphocytic leukemia. The authors found no significant effect of CYP3A5, ABCB1, ABCG2, and SLCO1B1 variants on ibrutinib levels; however, as an unexpected result, they found an association between the CYP3A4 *1/*22 genotype (a reduced-function variant) and a lower ADR incidence, suggesting a protective effect. This finding is unexplained and needs further confirmation in larger studies due to the limited statistical power of their study.

Mechanistic and evidence synthesis further broaden the ADR explanatory process. The review on cisplatin-induced ototoxicity (Contribution 8) emphasizes that some ADRs arise from complex, interacting biological pathways involving drug uptake, oxidative stress, mitochondrial injury, DNA damage, inflammation, and cellular vulnerability. In the review, the authors highlighted that prevention of hearing loss requires early identification of patients at risk and close clinical monitoring during treatment. Although classical TDM is not routinely established for cisplatin, the review supports the broader utility of TDM principles, particularly individualized dosing, consideration of cumulative exposure, renal function monitoring, and timely use of otoprotective strategies. The network meta-analysis of immune checkpoint inhibitor-associated pneumonitis illustrates another important methodology, namely, comparative evidence synthesis across randomized trials (Contribution 9). This approach quantifies ADR risks among agents within the same therapeutic class, comparing multiple treatments simultaneously, even if some of those treatments have never been compared head-to-head in a clinical trial. Such approaches move ADR research beyond the question of whether an event is reported more frequently and toward the question of why it occurs, in whom, and under which treatment conditions.

The third dimension of this collection concerns TDM and dose individualization. Over the last five decades, interest in TDM has increased, from less than 50 publications in 1970, including publications addressing ADRs and drug monitoring, to 233,701 publications today (PubMed search: 18 May 2026). Scientifically based TDM requires knowledge on the pharmacokinetics (PK) of the drug in question and information on the dose–therapeutic effect and dose–adverse-effect relationship. While more publications study the relationship between drug monitoring and therapeutic effect because the endpoint is clearly defined, it is more difficult to link drug monitoring to ADRs because of their widespread phenotypic diversity. Drug monitoring takes into consideration the variability and supports dose individualization. A prerequisite for an adaptive dose regimen is knowledge of the quantitative relationship between concentrations in biological fluids (blood, serum, plasma, urine) and the therapeutic outcome in terms of therapeutic success and of minimized adverse effects. The quantitative relationship between drug concentration and adverse effects was studied with the aim of providing optimal dosing and drug monitoring strategies in three publications (Contributions 10–12). Pharmacometrics were applied, including pharmacokinetic/pharmacodynamic modeling, population PK, and a prospective clinical observational study. In Contribution 10, the authors used physiologically based PK modeling and literature data of dose and adverse effects to clearly delineate the concentration cut-off for minor side effects and the cut-off for life-threatening side effects two hours after starting the infusion of 5-flurouracil. Hence, an individualized dose adjustment during the infusion is possible, illustrating the potential of TDM as an active intervention to prevent ADR. Similarly, the population PK study of piperacillin/tazobactam demonstrates how modeling and simulation can identify dosing regimens that better achieve pharmacodynamic targets across renal function groups and pathogen susceptibility levels (Contribution 11), thus shifting from reactive to proactive TDM. However, exposure-guided therapy in clinical practice remains complex. A meta-analysis on direct oral anticoagulants revealed inconsistent links between plasma concentrations and clinical outcomes, proving that drug concentration is merely one contributor of multifactorial risk. Because bleeding and thrombosis are heavily driven by age, renal function, and comorbidities, selective rather than universal TDM is most appropriate, particularly for vulnerable patients or uncertain clinical scenarios (Contribution 12). Conversely, monitoring TNF inhibitor concentrations during tapering helps predict flares, proving TDM can safely maintain efficacy while minimizing drug exposure (Contribution 13). Dose optimization of targeted anticancer agents further reinforces the need to move beyond fixed or historically established dosing schedules toward regimens that better balance benefit, toxicity, convenience, and patient burden (Contribution 14).

Finally, artificial intelligence emerges as a powerful tool for predicting ADRs, particularly by integrating chemical, pharmacological, mechanistic, and clinical data to assess complex drug–herb interactions (Contribution 15). Given that widespread, poorly documented herbal use frequently drives hidden toxicities, validated AI models offer an innovative way to prioritize interaction risks and guide clinical decisions.

The contributions to the Special Issue “Therapeutic Drug Monitoring and Adverse Drug Reactions” showcase a variety of approaches to address aspects of drug safety. They demonstrate that ADR research requires an integrated methodology. TDM and PK modeling can support individualized therapy but their success depends on well-defined exposure–response and exposure–toxicity relationships, while advancing TDM-guided safety requires addressing key translational obstacles. Research must expand beyond small molecules to characterize complex exposures in biologics, including target-mediated disposition and immunogenicity. Bedside translation further necessitates integrating PK models into user-friendly clinical decision-support tools and point-of-care testing. Incorporating patient-reported outcome measures will ensure that TDM addresses chronic, low-grade toxicities impacting adherence. Transitioning from static target ranges to Model-Informed Precision Dosing (MIPD) platforms, assisted by artificial intelligence, will move the field from reactive detection toward proactive ADR prevention.
